# Transformer‐based DNA methylation detection on ionic signals from Oxford Nanopore sequencing data

**DOI:** 10.15302/J-QB-022-0323

**Published:** 2023-10-17

**Authors:** Xiuquan Wang, Mian Umair Ahsan, Yunyun Zhou, Kai Wang

**Affiliations:** ^1^ Department of Mathematics and Computer Science Tougaloo College Jackson MS 39174 USA; ^2^ Raymond G. Perelman Center for Cellular and Molecular Therapeutics Children’s Hospital of Philadelphia Philadelphia PA 19104 USA; ^3^ Department of Pathology and Laboratory Medicine University of Pennsylvania Philadelphia PA 19104 USA

**Keywords:** Nanopore, long‐read sequencing, deep learning, Transformer model, DNA methylation.

## Abstract

Transformer is an algorithm that adopts self‐attention architecture in the neural networks and has been widely used in natural language processing. In the current study, we apply Transformer architecture to detect DNA methylation on ionic signals from Oxford Nanopore sequencing data. We evaluated this idea using real data sets (*Escherichia coli* data and the human genome NA12878 sequenced by Simpson *et al*.) and demonstrated the ability of Transformers to detect methylation on ionic signal data.

**Background:**

Oxford Nanopore long‐read sequencing technology addresses current limitations for DNA methylation detection that are inherent in short‐read bisulfite sequencing or methylation microarrays. A number of analytical tools, such as Nanopolish, Guppy/Tombo and DeepMod, have been developed to detect DNA methylation on Nanopore data. However, additional improvements can be made in computational efficiency, prediction accuracy, and contextual interpretation on complex genomics regions (such as repetitive regions, low GC density regions).

**Method:**

In the current study, we apply Transformer architecture to detect DNA methylation on ionic signals from Oxford Nanopore sequencing data. Transformer is an algorithm that adopts self‐attention architecture in the neural networks and has been widely used in natural language processing.

**Results:**

Compared to traditional deep‐learning method such as convolutional neural network (CNN) and recurrent neural network (RNN), Transformer may have specific advantages in DNA methylation detection, because the self‐attention mechanism can assist the relationship detection between bases that are far from each other and pay more attention to important bases that carry characteristic methylation‐specific signals within a specific sequence context.

**Conclusion:**

We demonstrated the ability of Transformers to detect methylation on ionic signal data.

## 1 INTRODUCTION

The complexity of the human genome and transcriptome lies not only in the composition of 3 billion base pairs, but also in the chemical modifications that make it interpretable to enzymes (writers, erasers, readers) through epigenetic regulation [1]. Genome‐wide epigenetic change, such as DNA 5‐methylcytosine (5mC), is a hallmark of cancer [2–4]. It is also widely known that 5mC methylation play important roles in brain development and function [5–9]. In addition to being diagnostic biomarkers in many diseases, DNA methylation is now a therapeutic target for cancer with several drugs being tested or approved by the US Food and Drug Administration [10,11]. For example, 5‐Aza‐2′‐deoxycytidine is among the first methylation inhibitor used in cancer clinical trials [12], and we demonstrated that it leads to isoform switching and exon skipping such as EZH2 in addition to de‐methylation [13]. Similarly, anti‐psychotic treatments have been linked to the alteration of DNA methylations [14], suggesting the potential of differential DNA methylation profiles as predictors of antipsychotic response.

Existing technologies, such as whole‐genome bisulfite sequencing and PacBio long‐read sequencing, have some inherent limitations to detect DNA modifications, because of the inability to detect modification in complex repetitive region, biases by incomplete and context‐dependent enzyme, and low signal‐to‐noise ratio [15,16]. Instead, Oxford Nanopore Technology (ONT) long‐read sequencing, which measures ionic current signals when DNA molecules translocate pores, may be a better option for the detection of DNA methylation. Recently, several analytic tools have been developed for DNA methylation detection on ONT long‐read sequencing data. The analysis methods can be generally classified into two types. One type of method compares raw signals of methylated DNA copies with signals of the un‐methylated DNA copies at specific genomic positions, such as Tombo/Nanoraw and NanoMod [17]. However, this method requires both methylated and un‐methylated samples that are available at the same time. Another type of method directly calls DNA modifications from ONT raw signals using machine learning approaches, such as Nanopolish [18], Megalodon [19], DeepSignal [20], Guppy [21], METEORE [22], and DeepMod [23]. For example, METEORE used random forest and multiple linear regression models, NanoPolish used hidden Markov model, and DeepSignal used convolutional neural network (CNN).

We previously developed DeepMod [23] which adopted a recurrent neural network (RNN) in a bidirectional LSTM architecture to detect DNA methylation from ionic signal data generated by ONT. We also recently released DeepMod2 that can handle moving tables generated by Guppy‐called or Tombo re‐squiggled data, since DeepMod requires event table used in older generations of data. In DeepMod, raw signals of each read are first translated into nucleotide sequences (basecalling). Signals are then aligned to corresponding reference nucleotides. After that, the target motif (*e.g.,* CpG) and its signals in a window of a fixed length are transformed into event‐based features as the input of methylation callers. Typical event‐based features include signal mean, signal standard deviation, event length, and nucleotide information, which tells the reference base in one hot encoding of the ACGT bases. The LSTM model bi‐directionally reads given nucleotide events sequentially (left‐to‐right or right‐to‐left). In DeepMod, we have released several pre‐trained models from different types of datasets including *Escherichia coli*, *Chlamydomonas reinhardtii* and human samples and demonstrated that it achieves good performance on these datasets. We note that Liu *et al.* comprehensively summarized current methods and made a comparison for human DNA methylation detection when comparing DeepMod with other tools such as Nanopolish and Tombo [24]. DeepMod’s DNA methylation detection was based on older basecallers (such as Metrichore), but other tools were based on Guppy basecalled data, therefore they are not comparable; instead, DeepMod2 should be used in this case.

Although DeepMod achieves good performance for different types of datasets according to several studies [22,25–27], we believe that the base modification problem is a language translation problem fundamentally and can be further improved. Since Transformers have proven to perform better than RNN and CNN in language modeling, here we leverage Transformers to identify modified DNA bases from signal data. Unlike LSTM, Transformer algorithms, such as BERT [28], do not necessarily process the input data in sequential order. Indeed, using DeepMod’s signal pre‐processing features, Zhang *et al.* further proposed MethBERT [25] utilizing a refined BERT method to detect DNA modification on ONT long‐read sequencing data.

Transformers adopt the mechanism of self‐attention, differentially weighting the significance of each part of the input data. The essence of the self‐attention mechanism is to detect the most useful information from many pieces of information. Just like the human brain, it will scan the entire field of view through the eyes, and then quickly locate the area of ​​interest. Then it devotes more attention resources to a specific area to obtain more detailed information about the target that needs attention, while ignoring other less useful information. This is a strategy by which humans use limited attention resources to quickly focus on high‐value information from a large amount of information. Multi‐head attention allows the model to jointly attend to information from different representation subspaces at different positions. Transformer’s self‐attention mechanism greatly improves the efficiency and accuracy of information processing.

In this project, we explored the use of a Transformer‐based BERT to further improve the methylation detection, and performed several experiments to answer the following questions: (1) how signal distribution differs in windows of varying sizes (such as 21 bp) surrounding methylated versus unmethylated cytosine, or in regions with enriched methylation (such as CpG islands) versus isolated methylation sites; (2) how the contextual signal changes with the length of the event window; (3) whether the number of heads in Transformer model will influence the accuracy of methylation detection.

## 2 RESULTS

### 2.1 Comparison of signal features for methylated and unmethylated sites in human genome

**Fig 1:**
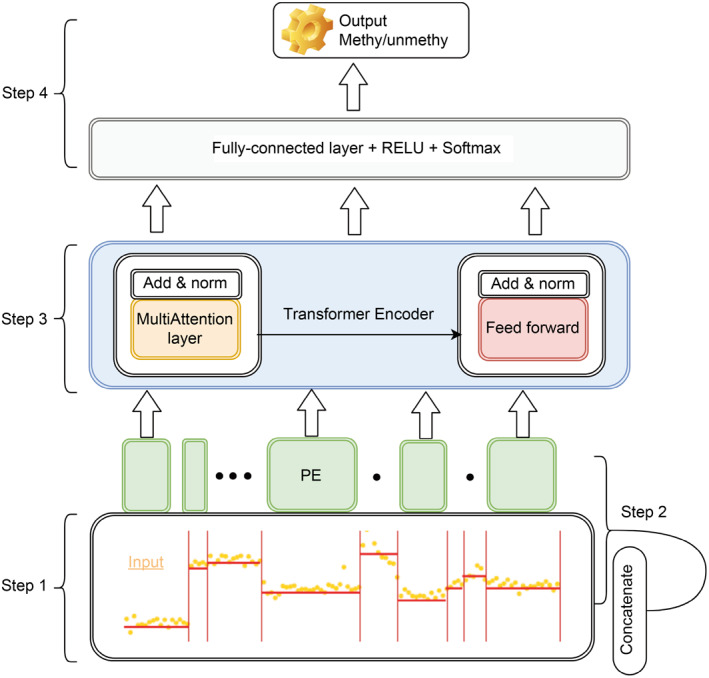
Architecture of the transformer‐based DNA methylation detection on ONT long‐read sequencing data.

**Fig 2:**
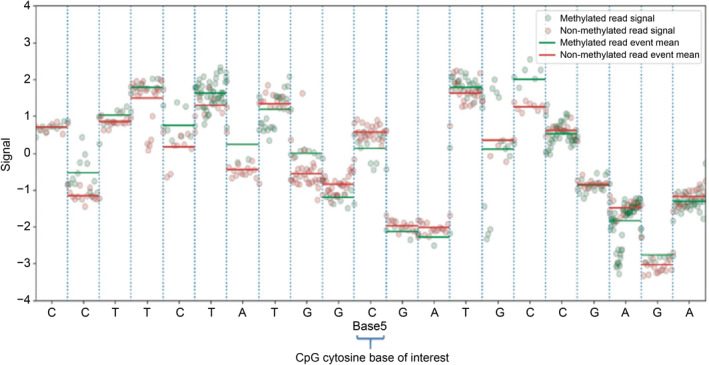
Signal visualization.

The general framework of our method is shown in Fig.1. Our method can directly detect DNA modification on ionic signals from ONT long‐read sequencing data based on the signal difference on methylated and unmethylated DNA. To give a visual demonstration, Fig.2 shows an example of how raw contextual signal is observed for 21‐bp window size on methylated and unmethylated location for CpG site at chr2:159219855 (GRCh38) on NA12878. This Fig.2 shows that the mean signal for methylated and un‐methylated location is different, and the CpG signal is influenced by its surrounding nucleotides.

### 2.2 Fine‐tuning of model parameters

**Fig 3:**
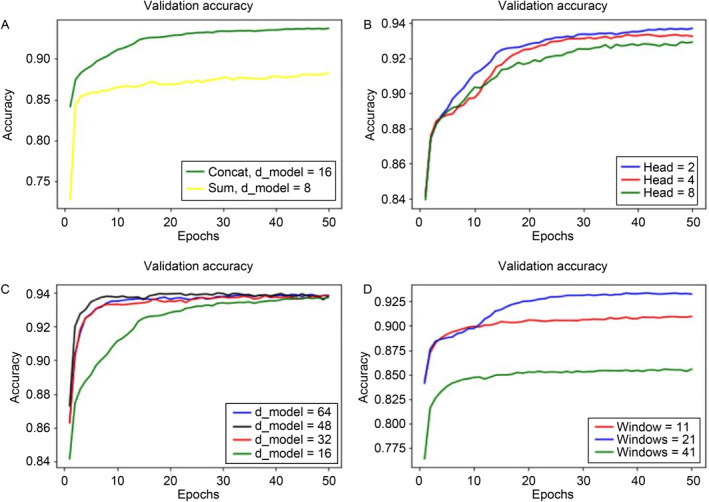
Experiments for model optimization.

To search for the best hyperparameters and optimize the model performance, we fine‐tuned the number of attention heads, the window size of events, and the feature size of each event. Simulation results of these hyperparameters tested on human genome NA12878 are shown in Fig.3. Detailed explanations for these experiments are given below.

#### 2.2.1 Performance with different position embedding (PE) strategy: summation vs. concatenation

The first experiment was to check the performance of the model on different position embedding method. We chose two different ways to combine our data feature vector and position embedding, *i.e.*, summation vs. concatenation, and we then ran 50 epochs on our validation data. We found that concatenation method was much better than summation on performance (Fig.3A).

#### 2.2.2 Performance with different number of attention head

We choose three attention head size (2, 4, and 8) to evaluate our model’s preformation (Fig.3B). Here, we set our d_model size to be equal to 16. We found that with 2 attention head, our model has the best performance. That is probably because 16 features represent a relatively small feature size; if we add more attention head to it, it may result in overfitting.

#### 2.2.3 Performance with different size of d_model

As mentioned above, when we implement the PE method, we found that the performance of concatenating PE vector with event vector is better than that of direct summation. However, the difference of these two methods may also be influenced by the size of d_model, *i*.*e*., feature size of each event vector. Therefore, we checked how the feature size of each event will affect the performance on our model. We start from feature size of 16 (concatenated 9‐feature position embedding to the original 7‐feature event), and continue to increase 16 more position embedding size each time. We found that the performance significantly improved during the first 15 epochs, and then converged to similar values at 50 epochs (Fig.3C).

#### 2.2.4 Performance with different window sizes

The last experiment we did was to check the model performance if we add more neighboring events on both sides of the event that we are interested in, *i*.*e*., given more contextual content by changing the window size. We found that 21 is the best window size to use in our model (Fig.3D).

After fine‐tuning the parameters to optimize the performance of the model, we suggest the values of the parameter at Tab.1.

**Tab 1:** Configuration of values for hyper‐parameter tuning

Parameter	Annotation of parameters	Type	Scope
d_model	Size of feature vector	Even integer	[32, 64]
Att. head	Number of attention heads	Integer	[27]
Att. layer	Number of attention layers	Integer	[27]
Batch size	Batch size	Integer	[256, 512]
Learning rate	Learning rate	Real	[0.001, 0.01]

Att. means attention.

### 2.3 Comparison of embedding patterns before and after concatenating PE

**Fig 4:**
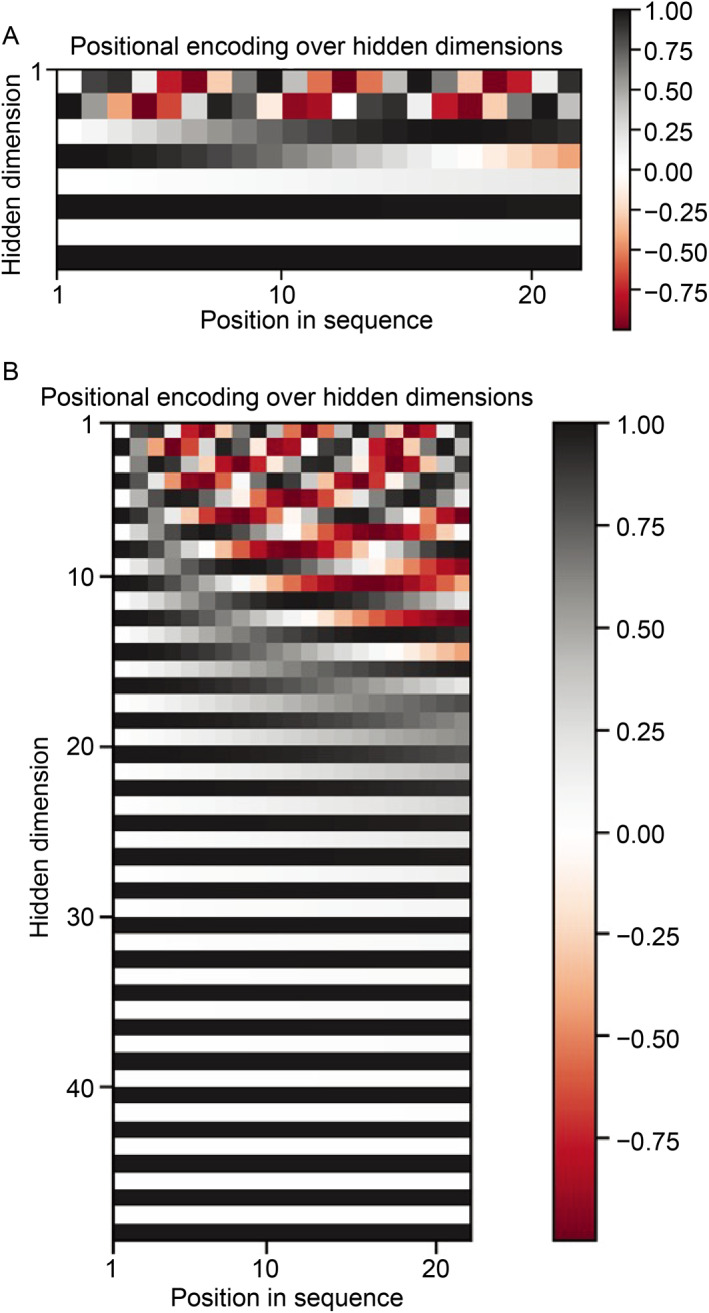
Embedding pattern visulization.

In order to assess how concatenating PE vectors influence the performance, we showed the embedding patterns before and after PE concatenation. When we choose 8 embedding vector size (Fig.4A), the pattern of the embedding is not clear: after we concatenate this embedding vector to the event features, the order of the event and the contextual relationship are not clearly seen. When we raise the embedding dimension to 48, we can easily see an increasing order of events based on the pattern on the hidden dimension (Fig.4B). After the hidden dimension is large enough to show the order of events, increasing dimension further will not improve the model performance.

### 2.4 Performance comparison across human genome and bacteria species

**Fig 5:**
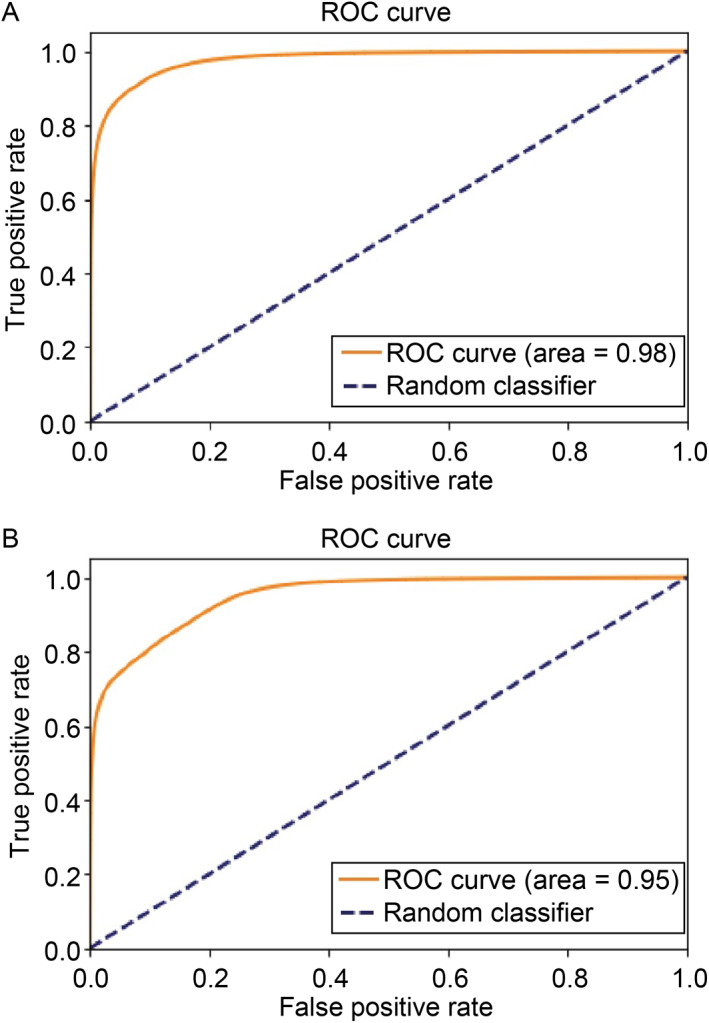
Performance evaluation.

We also performed cross‐species evaluation, and tested our model on human genome NA12878 and *E. coli* by training on one genome and testing on another. Fig.5 shows the ROC curves for 5mC detection on both species.

As we can see from the Fig.5, our method achieves good performance on both *E. coli* (AUC = 0.99) and NA12878 (AUC = 0.95). We further compared the performance with DeepMod and DeepSignal. We found that DeepMod achieves higher F1 scores on both human and bacteria genomes than the other two methods, as shown in Tab.2.

**Tab 2:** Performance of Transformer‐based method comparing with DeepMod and Deepsignal on NA12878 and *E. coli*

Dataset	Models	F1 score
NA12878	Transformer method	0.8600
	DeepMod	0.8706
	deepsignal	0.8520
*E. coli*	Transformer method	0.9410
	DeepMod	0.9501
	deepsignal	0.9330

## 3 CONCLUSION AND DISCUSSION

In the current study, we propose a Transformer‐based method for detecting DNA methylations from ionic signal data generated by Nanopore sequencing. We begin with the hypothesis that the performance of 5mC prediction may be improved using more sophisticated deep neural networks—Transformer. Then by modifying the encoding part of the Transformer, we can capture the difference of properties between methylated and un‐methylated bases. Our results demonstrate preliminary success of Transformers in detecting methylations, but the current model did not perform optimally yet (for example, DeepMod outperformed the current transformer model). We stress that this is an exploratory study to see whether Transformer may work on methylation detection, since the methBERT study did not evaluate different model architectures (such as position embedding strategies) comprehensively.

We believe that DNA methylation detection closely resembles language translation because of both short‐term distance dependency and long‐term distance dependency. When DNA strands translocate Nanopore, approximately 7 nucleotides are covered within the pore, so they had the greatest contribution to the signal patterns. However, adjacent nucleotides, before or after the translocation, as well as the sequence contexts (such as the formation of secondary structure or location within a specific sequence motif), also determines the signal patterns in Nanopore sequencing. Therefore, such relationships closely resemble language translation, which we believe can be addressed by employing methods used in NLP tasks.

In the past few years, the field of NLP is revolutionized by the use of various Transformer models. Transformers, such as BERT, is able to achieve better performance than RNN [29] and enable parallel computing with faster running speed. Parallel processing is particularly useful for processing Nanopore signals, because it can speed up the prediction of modifications from Nanopore signals on large data set from PromethION flowcells (>1 TB/flowcell). Additionally, self‐attention in Transformer can efficiently capture long‐range dependencies, which is a critical issue that RNN may not address well.

To the best of our knowledge, only one study (MethBERT) used BERT model to detect DNA methylation from ONT data. MethBERT utilized the DeepMod framework to perform the same pre‐processing for the electrical raw signal data and then used a refined BERT model as a core (instead of LSTM) to detect DNA methylation. The refined BERT uses learning PE and relative position representation. The learnable PE takes positional embedding vectors as parameters, which are updated during the learning process. Their experiment show that the refined BERT can achieve competitive and even better results than the state‐of‐the‐art bidirectional recurrent neural network (bi‐RNN) model on a set of 5mC and 6mA benchmark datasets while the model inference speed is about 6x faster.

Similar to DeepMod’s approach using a bi‐RNN, Transformers implements the concept of two‐way signal processing as well, but are more efficient in parallelization. Based on our experiments, Transformer‐based method did not significantly outperform DeepMod’s LSTM method for both human and bacteria genome. We should note that the performance of a model is dependent on the task and the data, and these factors should be taken into consideration when choosing a model. Additional improvements in this field requires innovations that directly assay signals (without basecalling), rather than two‐step deep learning‐models. In the future, we plan to improve the model from the following perspective: (1) computational speed; (2) how we embed the signal; (3) generate more training samples.

## 4 METHODS

We developed a Transformer‐based method for the detection of DNA modifications from Nanopore sequencing data. The Nanopore sequencing dataset we used for training and testing for 5mC detection include *E. coli* data and the human genome NA12878 sequenced by Simpson *et al*. [18]. The human genome NA12878 has been well‐studied with various sequencing data, including Nanopore, PacBio, Illumina bisulfite sequencing with two replicates, and methylation microarrays.

As the framework of our method shown in Fig.1, it consists of four steps: anchoring signals to reference positions after read alignment, feature generation, modification prediction via Transformer model, and genome‐scale modification summary.

Step 1: The input includes a reference genome and FAST5 files containing raw signals and events (nucleotide, A, C, G or T), which were generated by Nanopore sequencers with base calls. Each event is then encoded in one‐hot form in the order of (ACGT) and was represented with a 4‐feature vector (*e.g.,* C:=<0,1,0,0>). In this 4‐feature vector, 1 indicates that the mapped reference base is a specific nucleotide type C, whereas 0 means otherwise. Raw signals for all aligned bases in a long read were normalized and rescaled from −5 to 5. Then the signal mean, standard deviation, and the number of signals associated with an event were extracted. Thus 7‐feature vector was obtained $x_{i}=<f_{m},f_{sd},f_{l},f_{A},f_{C},f_{G},f_{T}>$.

The self‐attention heads (attention modules) can learn contextual relations between nucleotides. Therefore, for the interested event $x_{i}$, we take its $\left\lfloor \frac{w}{2}\right\rfloor$ upstream events and $\left\lfloor \frac{w}{2}\right\rfloor$ downstream events into consideration as context, *i*.*e*.

$$x=<x_{i-\frac{w}{2}},\text{…},x_{i},\text{…},x_{i+\frac{w}{2}}>$$

By default, *w* = 21, but this is a parameter that can be changed by users.

The problem with the event sequence x is that it only records the base type and some basic statistical information of each event, but does not record the position information of these events in the sequence. We know that when the same event appears in different position in the sequence, its function or signal characteristics may be completely different. So, we should also record the position information of the event in the sequence, which is the goal of position embedding.

Step 2: Position embedding was first proposed by Vaswani et al. in 2017 [29]. It was originally used in NLP and added after the word vector layer to supplement position information. In our project, we compared two embedding methods: Adding vs. Concatenating. We then choose the concatenating method based on the performance of our experiments, *i.e.,* we let $x_{i}=<f_{m},f_{sd},f_{l},f_{A},f_{C},f_{G},f_{T},f_{pe_{1}},\text{…},f_{pe_{n}}>$, to capture the position information, where $f_{pe_{j}},j=1,\text{…},n$ are position vector created with the Sinusoidal PE (Eq. (1))

(1) $$x_{\left(i,2j\right)}=sin\left(i*w_{0}^{\frac{2j}{d_{mod}}}\right),\;\;x_{\left(i,2j+1\right)}=cos\left(i*w_{0}^{\frac{2j}{d_{mod}}}\right),$$

where i is the position of the event and $w_{0}=1/10,000$ is the minimum frequency of the embedding. $d_{mod}=n+7$, the dimension of the $x_{i}$, which is also known as the dimension of the model, must be an even integer and a multiple of the number of attention heads.

This feature‐based event vector with size [wby(n+7)] was used as input of the following Transformer encoder module to predict whether the center event of the w events is generated from a modified base.

Step 3: The Transformer encoder module follows overall encoder layer of Transformer architecture using stacked self‐attention and point‐wise, fully connected layers to capture complicated relationship between signals and prediction target of modifications.

Given a long read with sequential events, an interested event $x_{i}$ with dimension (n+7) and its neighborhood $<x_{i-\lfloor \frac{w}{2}\rfloor },\text{…},x_{i},\text{…},x_{i+\lfloor \frac{w}{2}\rfloor }>$ could be used as input of this Transformer encoder. We then pass these vectors to the “self‐attention” layer. Self‐attention uses the attention mechanism to calculate the association between each event and all other events to get attention score. Using these attention scores, a weighted representation can be obtained, which is an equivalent list of (*n*+7)‐dimension vectors, and it is then fed into the feedforward neural network to obtain a new representation, which takes contextual information into account. The output of the feedforward neural network is also a list of (*n*+7)‐dimension vectors, and then the output is passed up to the next encoder layer. This process can be repeated for events of interest in a long read, and then for all long reads that are available for analysis. Each layer has two sub‐layers. The first sub‐layer represents a multi‐head self‐attention mechanism, and the second sub‐layer is a simple, position‐wise fully connected feedforward network.

Step 4: The output of the Transformer encoder is a list of vectors of floats. The modification for the interested event is predicted by the final fully connected network, ReLu activate function, and softmax layer. Suppose our model trains a total of 10,000 interested events from the training dataset in the experiment. The corresponding output vectors from Transformer encoder was sent to the final fully connected layer to generate a vector with 10,000 dimensions, each representing the score of an interested event. After the linear layer there is a softmax layer that converts these scores into probabilities. We then identify the interested event with probability higher than 0.5 as methylation nucleotides and events with probability equal to or lower than 0.5 as unmethylation nucleotides, and then use these predictions as the output for the downstream analysis.

### 4.1 Training and testing process

We train the model on the *E. coli* datasets, and then test it on human genomes in order to demonstrate the feasibility of using our method on different species as we did for DeepMod. In detail, given a training set: $D={\left\{\left(x_{i},y_{i}\right)|x_{i}\in R^{d_{mod}},y_{i}\in \left\{0,1\right\}\right\}}_{i=1}^{N_{total}}$, we assume that $y_{i}=1$ for methylation bases while $y_{i}=0$ for un‐methylation bases. For each input event $x_{i}$, it outputs a predicted value $H\left(x_{i}\right)$. Then the predicted value will be compared with the actual label $y_{i}$, we will get the following four situations:

• the prediction is positive, and the actual is also positive, we call it true positive (TP),

• the prediction is positive, the actual is negative, we call it false positive (FP),

• the prediction is negative and the actual is positive, which is called false negative (FN).

• the prediction is negative and the actual is also negative, which is called true negative (TN).

If we define a test set, the number of completely modified bases is P and the number of completely unmodified bases (or motifs of interest) is N, then Accuracy, Precision, Recall, and AUC are used to evaluate the performance.

(2) Accuracy=(TP+PN)/(P+N)

(3) Precision=TP/(TP+FP)

(4) Recall=TP/P

(5) F1=(2∗Precison∗Recall)/(Precision+Recall)

## 5 DATA AND CODE AVAILABILITY

All code for data cleaning and analysis associated with the current submission is available upon request.

## 7 COMPLIANCE WITH ETHICS GUIDELINES

The authors Xiuquan Wang, Mian Umair Ahsan, Yunyun Zhou and Kai Wang declare that they have no conflict of interest or financial conflicts to disclose.

This article does not contain any studies with human or animal materials performed by any of the authors.

## 8 OPEN ACCESS

This article is licensed by the CC By under a Creative Commons Attribution 4.0 International License, which permits use, sharing, adaptation, distribution and reproduction in any medium or format, as long as you give appropriate credit to the original author(s) and the source, provide a link to the Creative Commons licence, and indicate if changes were made. The images or other third party material in this article are included in the article’s Creative Commons licence, unless indicated otherwise in a credit line to the material. If material is not included in the article’s Creative Commons licence and your intended use is not permitted by statutory regulation or exceeds the permitted use, you will need to obtain permission directly from the copyright holder. To view a copy of this licence, visit http://creativecommons.org/licenses/by/4.0/.
